# Microsatellite development for *Theridion evexum* (Araneae: Theridiidae) using low-coverage genome sequencing and the MiMi script

**DOI:** 10.1371/journal.pone.0331200

**Published:** 2025-09-15

**Authors:** Ruth Madrigal-Brenes, Gilbert Barrantes, Luis Sandoval, Eric J. Fuchs

**Affiliations:** 1 Centro de Investigación en Biodiversidad y Ecología Tropical, Universidad de Costa Rica, San José, Costa Rica; 2 Escuela de Biología, Universidad de Costa Rica, San José, Costa Rica; 3 Laboratorio Binacional de Análisis y Síntesis Ecológica, UNAM-UCR, México, Costa Rica; 4 Laboratorio de Ecología Urbana y Comunicación Animal, Escuela de Biología, Universidad de Costa Rica, San José, Costa Rica; Central University of Punjab, INDIA

## Abstract

Habitat fragmentation and unplanned urban expansion increasingly threatens biodiversity, yet the genetic impacts on arthropods, and particularly spiders, remains understudied due to the lack of suitable molecular makers. Here, we used low-coverage genome sequencing and a bioinformatics pipeline to develop polymorphic microsatellite markers for *Theridion evexum*, a tropical spider with urban and natural populations. To increase the yield of DNA extracted from small spiders like *T. evexum*, we also optimized a Cetyltrimethylammonium bromide (CTAB) DNA extraction protocol. We sequenced eight individuals at 4X using paired-end sequencing on an Illumina Novaseq 6000. Reads were cleaned and processed using the Multi-individual Microsatellite Identification (MiMi) python pipeline. MiMi produced a total of 3999 putative microsatellites. After filtering for polymorphic loci with an allelic richness greater than three and present in at least 5 of the 8 sequenced individuals, 34 final markers were identified. An experimental validation of 13 of these 34 markers showed that 10 loci were polymorphic with at least three detectable alleles, one locus was monomorphic, and two loci did not produce PCR products. These markers will allow a better assessment of the effects of urban fragmentation and isolation across populations of this spider species. Furthermore, developing markers using low-coverage next-generation sequencing (NGS) and bioinformatic protocols provide a valuable approach for uncovering microsatellite markers at a reduced cost for other tropical species, thereby broadening the scope of molecular ecology research in the tropics.

## Introduction

The effects of urban expansion and land-use changes on natural habitat fragmentation and loss have been primarily studied in vertebrates and plants, with a focus on genetic structure and gene flow [1–3]. Arthropods, such as spiders, have received limited attention, and the majority of studies prioritize species diversity over genetic diversity [4,5]. The lack of specific genetic markers for tropical species limits the study of the impacts of fragmentation and habitat degradation on genetic diversity and gene flow among isolated habitats. Understanding the underlying processes of urban expansion on the genetics of tropical spiders is critical for assessing their resilience and adaptability, particularly in increasingly fragmented settings.

Spiders are a highly diverse group of arthropods that play a significant role as predators in various ecosystems, including those in urban areas. The limited dispersal ability and specialized microhabitats of some tropical spiders make them highly susceptible to the drastic landscape transformations caused by urbanization [6–8]. In urban environments, isolated vegetation patches can increase genetic structure and genetic drift in various organisms, likely affecting their adaptability and individual fitness. Although similar information for tropical spiders is lacking, genetic isolation may have severe effects, particularly for specialized species [6,9–11], whose populations tend to decline more rapidly as urbanization intensifies.

Molecular markers have been used in spiders to elucidate evolutionary relationships (phylogenetic relationships) among different taxa [12–14]. Despite their potential, microsatellite markers have been developed for only a very limited number of tropical species, limiting studies of genetic diversity and gene flow [15–17]. The effectiveness and transferability of these markers to other species is still unknown, which limits their potential applications in genetic structure and diversity studies.

Microsatellites, also known as simple sequence repeats (SSRs), are short tandemly repetitive DNA sequences, exhibiting a high degree of polymorphism in the number of repeats. SSRs are distributed throughout the genomes of both prokaryotes and eukaryotes [18,19]. Given their high diversity, Mendelian inheritance, and repeatability, SSRs have been widely used to study the genetic diversity of multiple species [20,21]. However, one of the major problems associated with SSRs is their high degree of specificity, as their location in the genome varies significantly between species. Therefore, traditional molecular techniques to identify SSR and develop PCR primers for these loci across different species are often complex and expensive [22,23]. The recent development of next-generation sequencing (NGS) and the possibility to sequence low-coverage genomes have prompted the creation of bioinformatics protocols to discover SSRs from genomic data [24–26]. These protocols allow the discovery of SSR markers in unstudied species without incurring the high costs and technical difficulties of previous protocols, which rely on enriched libraries of repeated sequences and hybridization protocols, frequently exceeding the US$ 10,000 mark [23]. However, many of these protocols rely on single individuals or pooled, untagged reads from multiple individuals [24,27,28], which may limit the ability to select markers with reliable levels of polymorphism because it becomes difficult to assess if different alleles are present in different individuals.

This study aims to detect microsatellite markers and design suitable PCR primers to amplify these markers for the tropical spider *Theridion evexum* (Theridiidae) using low-coverage whole genome sequencing (lcWGS) and the MiMi Python script [25]. We will also experimentally test a subset of these primers on individuals from natural populations to assess their polymorphism and their potential to provide informative estimates of genetic diversity and structure in populations of this species. This study will evaluate the potential of low-coverage whole genome sequencing and the MiMi script as a cost-effective and efficient method for developing microsatellite markers, particularly for understudied species in low- and middle-income countries. This research has broader applications for studying the impacts of urbanization on biodiversity and developing molecular tools for conservation and management.

## Materials and methods

### Study species

*Theridion evexum* is a spider native to the American tropics. In Costa Rica it is found from 200 to 1700 m asl with the highest abundance between 1200 and 1500 m. Its distribution is associated with permanent streams (approximately 25 m from the stream margin). This spider builds shelters by folding a leaf into a cone shape and spinning a capture web that consists of sticky threads running downward to adjacent leaves [29]. In Costa Rica, they build their webs in the understory of humid forests.

### Sample collection and DNA extraction for SSR identification

We collected a total of 16 individuals of *T. evexum*, with four individuals sampled from each of four distinct populations: University of Costa Rica (UCR) (9°56′ N, 84°03′ W), Lankester Botanical Garden (JBL) (9°50′ N, 83°53′ W), Parque del Este (PE) (9°56′ N, 84°00′ W), and Cascajal de Coronado (CC)(10°03′ N, 83°56′ W). Individuals were preserved in 95% alcohol and processed at the Laboratorio de Ecología Molecular (LEM) at Universidad de Costa Rica. We photographed and deposited one voucher specimen from each population at the Museo de Zoología, Universidad de Costa Rica.

The abdomen of the spider was removed and discarded, leaving the cephalothorax and legs, which were placed on a paper towel for 5 minutes to allow excess alcohol to evaporate. The cephalothorax was then transferred to a pre-chilled 1.5 mL microcentrifuge tube (cooled in liquid nitrogen for 15 seconds), and approximately 1 mL of liquid nitrogen was added to freeze the sample completely. The tissue was ground into small pieces (< 1 mm) or a fine powder using a plastic pestle. Subsequently, 500 µL of cetyltrimethylammonium bromide (CTAB) buffer (2% CTAB – 100 mM Tris-HCl, 1.4 M NaCl, 20 mM EDTA, 2% (m/v) CTAB, 2% (v/v) PVP) and 20 µL of proteinase K were added. The samples were incubated overnight at 55°C with continuous shaking at 800 rpm.

After incubation, the samples were centrifuged. Approximately 400 µL of the supernatant were carefully transferred to a labeled 2.0 mL tube. To this, 400 µL of Chloroform-Octanol (24:1) was added, and the tubes were mixed by inverting them for 2 minutes. The mixture was then centrifuged. From the supernatant, the maximum volume without disturbing the organic phase was transferred (~300 µL) to a labeled 1.5 mL tube, followed by the addition of 300 µL of isopropanol cooled to −20°C, mixed by inverting for 2 minutes, and subsequently refrigerated at −20°C for one hour. The tubes were centrifuged, and the supernatant was carefully discarded, without disturbing the pellet. Subsequently, 500 µL of 70% ethanol was added, mixed by vortex for five seconds and followed by centrifugation and decantation. This ethanol wash was performed twice, and the tubes were then left upside down on a napkin to remove excess ethanol. Once the pellet was completely dry, it was eluted in 50 µL of TE. All the centrifugation steps were performed at 14,000 rpm for 5 minutes.

DNA concentration and quality were measured using the Quantus fluorometer (Promega) and NanoDrop 2000 spectrophotometer (Thermo Scientific), respectively. We considered DNA to be of high quality (quality criteria) if concentrations were close to or above 20 ng/mL in the fluorometer and absorbance ratios in the spectrophotometer of 260/280 were close to 1.8–2.0 and 260/230 were between 2.0 and 2.2. We have uploaded this optimized DNA extraction protocol to protocols.io: https://dx.doi.org/10.17504/protocols.io.kxygxwp2kv8j/v1 and it is included as a supplemental file S1 File. This optimization step was necessary because *T. evexum* is a small-bodied spider, which may limit the amount and quality of DNA extracted for NGS.

### Next-generation sequencing

From the 16 extracted individuals, we selected DNA from eight samples (two from each population) that had the highest values in our previously defined quality criteria. We sequenced these samples to an approximate coverage of 4 Gb per individual at Novogene in South Korea. Although information on genome size for *T. evexum* is unavailable, reports from other species in the Theridiidae family indicate that genomes range from 1.3 to 2.1 Gb [30]. We performed paired-end sequencing on a NovaSeq 6000 using the Illumina platform, following Novogene’s corporate standards.

### Bioinformatic analysis and SSR discovery

To detect microsatellite markers for *T. evexum*, we used the Python script “Multi-individual Microsatellite Identification (MiMi)” [25] (https://github.com/graemefox/mimi). MiMi enables comparing microsatellites from multiple individuals simultaneously, improving the identification of loci with a higher probability of amplification. It also provides observed polymorphism estimates, allowing for a more accurate selection of SSRs.

#### Quality control.

We initially performed quality control using Trimmomatic version 0.38.1 as implemented in the Galaxy platform [26,31] to eliminate low-quality reads. We removed low-quality bases and Illumina adapter contamination using Trimmomatic with the following parameters: a sliding window of 4 bases requiring a minimum average quality score of 30 (SLIDINGWINDOW:4:30), removal of low-quality bases from the start and end of reads with a quality threshold of 3 (LEADING:3; TRAILING:3), and discarding reads shorter than 50 bp after trimming (MINLEN:50). Illumina adapter sequences were identified and removed with a minimum match length of 8 bases to ensure specificity while minimizing false positives.

#### Microsatellite identification.

As a second step, we used the software *pal_finder* [32] version 0.02.04 deployed on the Galaxy server (https://palfinder.ls.manchester.ac.uk) to identify microsatellite loci on each individual separately. For *pal_finder*, we maintained all default options as suggested by Griffiths et al. [26], except for the minimum *Phred* quality of the sequences used to detect microsatellites, which we increased from 20 to 30 to improve the reliability of microsatellite detection [33]. The SSR sequences generated by *pal_finder* were transferred to the KABRE High Performance Computer (HPC) at the Centro Nacional de Alta Tecnología (CeNAT) in Costa Rica. In the KABRE HPC, we ran the MiMi Python script version 1.1 on the SSR sequences generated by *pal_finder* using the default settings for MiMi.

#### Microsatellite filtering.

The MiMi results include an estimate of the observed number of alleles per marker and individual. MiMi calculates the number of alleles based on the number of tandem repeats identified [25] and considers each unique tandem repeat length as a distinct allele. We used these estimates to filter the initial set of microsatellites discovered by MiMi, choosing those markers with more than three alleles and requiring their presence in a minimum of six of the eight sequenced individuals (S1 Table). We uploaded this bioinformatics protocol to protocols.io: https://dx.doi.org/10.17504/protocols.io.dm6gp9bo8vzp/v1, and we included it as supplemental file S2 File.

### Experimental test of SSR

The MiMi pipeline successfully identified 34 candidate microsatellite markers through *in silico* analysis. To determine whether the designed primers could reliably amplify across different individuals, we chose the 13 most polymorphic loci (i.e., more than three alleles) that were present in at least 75% of the samples (6 out of 8 sequenced individuals) for experimental testing on field-collected samples (S1 Table). Primers were labeled with the fluorescent dyes 5’-FAM and 5’-HEX for genotyping (Table 1). For the experimental test, we collected and extracted DNA from 15 new individuals per population from both CC and UCR for a total of 30 individuals. DNA extraction was conducted using the CTAB protocol described above.

**Table 1:** Experimentally evaluated primers in a sample of 30 individuals from two *T. evexum* populations in Costa Rica.

Locus ID	Repeat motif	Forward primer sequence (5’-3’)	Reverse primer sequence (5’-3’)	5’ Dye	Size range (bp)
Thev01	AT	TGTTAATGCTGAAAGACGCC	TTGCTGAAGGGTATCTTAACGC	FAM	197-205
Thev02	ATT	ACATTTCAGTGCTTCCAGTAACC	AGCAAATGCTGTGTTCTGCC	HEX	208
Thev03	TC	TGCACCAATAAAAGGGGTCC	TCAGGATCAGACCATTTCAGG	FAM	*
Thev04	AGT	TTTCCGTATTCAACTACTTAGCCG	CGGAATTTGTTTTGGTTCGG	HEX	231-247
Thev05	ATT	CTTGACCGTATTGCGCAGC	GTCTTGTGCATTGCATTCCC	FAM	*
Thev06	AC	TTGCGACCCTTGTAAAGACC	TATGTAGGATGTTCCCTTTTGC	HEX	141-175
Thev07	AC	TCTGAGTTTCTCAAATCAACCCC	CCAGGGCTCATATCTTATTCTTGC	FAM	288-310
Thev08	AT	CGAGATGTTTAGCTCCTTCTGC	GAATATCGTTTTCTCCGCCC	HEX	274-300
Thev09	TC	CCCTTAGGCCAACTAACCTCG	TGCATTGGAGAAAACTTTCGG	FAM	230-270
Thev10	TC	CTTATGCAATGGGAAAGGGC	ATTTATTCCCGGTTCCTCCC	HEX	214-236
Thev11	AT	CTCGTGCAACGAAAATGAGG	GGTTACGATCCAACCCTGC	FAM	220-230
Thev12	TC	TGCAGTTGCTACGCTACAGG	GAGTTATTTTCGTGGAAGCCG	HEX	118-146
Thev13	AT	TTAAGTTTGGAGAACGGGGC	TGCATTAGGACCGGCATAGC	FAM	227-243

*Thev03 and Thev05 did not amplify and thus have no size range.

We initially checked PCR amplification for these markers in simplex reactions using 2X GoTaq® G2 Hot Start Master Mix (Promega), with 0.2 μM primers in a total reaction volume of 12.5 μL, incorporating 10 ng of DNA template. The PCR profile included a denaturation step at 95°C for 60 seconds, followed by annealing at 53°C for 60 seconds and extension at 72°C for 60 seconds, repeated for a total of 30 cycles, with a final extension at 72°C for 5 minutes, performed on a Bio-Rad T100 Thermal Cycler.

Two markers, Thev03 and Thev05, failed to yield PCR products, while the remaining eleven markers amplified successfully, with ten polymorphic loci (Thev02 was monomorphic). We then created three multiplex mixes to genotype the 30 individuals for an initial assessment of genetic diversity: Mix-1 included Thev01, Thev04, and Thev06; Mix-2 included Thev07, Thev09, and Thev10; and Mix-3 contained Thev08, Thev11, and Thev12. We analyzed Thev13 in a simplex reaction because when we included it in Mix 1 its electropherograms were unclear and difficult to genotype. Both multiplex and simplex reactions were performed using the same PCR conditions.

For fragment analysis, we used an Applied Biosystems ABI 3500 automated sequencer. We manually scored alleles using GeneMarker Software version 2.6.4 (SoftGenetics). We assessed the probability of null alleles and allelic dropout with the software *Micro-Checker* 2.2.3 [34] using default settings. To estimate genetic diversity, we used the *poppr* and *adegenet* libraries in the R statistical environment [35]. We calculated an observed allelic richness (Ar), effective number of alleles (Ae), observed (Ho) and expected heterozygosites (He), and the inbreeding coefficient (F). Significance values for inbreeding coefficients were calculated using FSTAT version 2.9.3.2.

### Ethics statement

All samples collected in this study received approval from the Biodiversity Commission of the Universidad de Costa Rica, under collecting permit number N° 127600002134.

## Results

We obtained a mean of 46.3 ng/μL ± 19.6 (mean ± SD) of DNA of high quality and purity from the eight individuals sent for next-generation sequencing. The recovered DNA had a mean absorbance of 2.0 (range: 2.0–2.1) at 260/280 and 2.1 (range: 2.0–2.3) at 260/230. Novogene generated a total of 35.9 Gb of raw data with a mean coverage of 1.3 Gb (± 0.18 Gb). Sequence effectiveness was on average 97.91% (± 1.21%), with an error rate of 0.03% for all samples. The percentage of sequences that had *phred* scores ≥ 30 was 90.46% (±0.74%), and the average GC content of all sequences was 30.91% (±0.27%).

### SSR discovery

MiMi discovered 3999 possible microsatellites. Of these 3999 SSRs, 555 were polymorphic with more than one allele by MiMi’s standard (i.e., set of tandem repeats, see Methods). Testing 555 SRR markers is economically unfeasible, so we selected the most polymorphic loci and those most likely to amplify (markers present in 62.5% of the samples, number of alleles > 3), resulting in 34 microsatellites (dinucleotides: 25, trinucleotides: 5, tetranucleotides: 4). The number of microsatellite markers and their repeat motif in each filtering step is shown in S1 Table. Primer sequences (forward and reverse), *in silico* number of alleles, and allele repeat motif for the selected 34 microsatellite loci are shown in S2 Table.

### Genetic diversity results of experimental validation

The number of alleles per polymorphic locus varied from 2 (Thev01) to 9 (Thev07), with a grand mean of 4.65 ± 0.386 (SD) across both populations. Only Thev02 was monomorphic. The observed heterozygosity ranged from 0.133 to 0.846, with a mean across populations of Ho = 0.478 ± 0.137. Expected heterozygosity had a similar range from 0.250 to 0.881, with a mean of He = 0.604 ± 0.152. Diversity estimates for each locus and population are shown in Table 2. After correcting for multiple comparisons, only locus Thev11 at UCR was not in Hardy-Weinberg equilibrium, indicating a significant heterozygote deficit. Similarly, MICROCHECKER only found evidence of null alleles in locus Thev09 in the same population. Across loci, both populations showed significant inbreeding coefficients (Table 2), and the overall estimate for pooled populations (may include Wahlund effect, Nei’s G_ST_ = 0.08, p < 0.01) was F = 0.226 ± 0.303, suggesting a general deficit of heterozygotes.

**Table 2:** Genetic diversity estimates for each locus in the two populations of *T. evexum* in Costa Rica.

Pop	CC					UCR				
Locus	A	Ae	Ho	He	F	A	Ae	Ho	He	F
Thev01	2	1.867	0.200	0.491	0.592*	3	1.587	0.154	0.394	0.610*
Thev04	3	2.273	0.600	0.579	−0.037 ^NS^	5	1.798	0.308	0.468	0.343 ^NS^
Thev06	5	3.462	0.667	0.738	0.097 ^NS^	5	2.153	0.615	0.555	−0.110 ^NS^
Thev07	5	2.695	0.667	0.650	−0.026 ^NS^	9	6.500	0.846	0.881	0.040 ^NS^
Thev09	6	3.462	0.200	0.755	0.735***	3	2.181	0.154	0.580	0.735***
Thev10	7	3.947	0.800	0.771	−0.037 ^NS^	6	2.914	0.692	0.683	−0.014 ^NS^
Thev08	3	1.312	0.133	0.250	0.467 ^NS^	6	3.485	0.539	0.750	0.282 ^NS^
Thev11	3	2.528	0.533	0.629	0.152 ^NS^	3	2.889	0.231	0.699	0.670 ^NS^
Thev12	5	2.813	0.667	0.667	0.000 ^NS^	6	2.380	0.615	0.603	−0.021 ^NS^
Thev13	4	1.636	0.333	0.405	0.177 ^NS^	4	2.099	0.615	0.542	−0.136 ^NS^
Mean	4.3	2.599	0.480	0.593	0.212**	5.0	2.799	0.477	0.615	0.240**

Ar: allele number, Ae: effective number of alleles, Ho: observed heterozygosity, He: expected heterozygosity, F: inbreeding coefficient. *: p < 0.05; **: p < 0.01; ***: p < 0.001; ^NS^: F is not significantly different from zero.

## Discussion

In this study, we successfully identified primers for microsatellite loci in the tropical spider *Theridion evexum* using low-coverage genome sequencing and the MiMi Python script. This is a cost-effective and simple approach for microsatellite discovery, which is suitable for understudied taxa such as tropical spiders. Traditional techniques that require library enrichment of repeated sequences are costly and time-consuming, often resulting in highly specific SSR primers. In contrast, our study demonstrates that microsatellite loci can be identified directly from low-coverage genome sequence data. Given the rapid advancements in next-generation sequencing technologies and the decreasing costs of data generation, we believe that this approach will become a useful tool for microsatellite development, especially in resource-limited countries where SNP-based studies continue to be expensive. These primers also have the potential to cross-amplify in related species, increasing the potential benefits of using the technique presented in this study.

Our findings reveal that the modified CTAB protocol we used produced sufficient amounts of DNA. *T. evexum* is a relatively small species, characterized by slender legs and small cephalothoraxes; therefore, the amount of DNA obtained is rather low. High-quality DNA is necessary for NGS sequencing, which could be a problem when working with small individuals like *T. evexum*. This problem is exacerbated by the limited number of adults present at any time in natural populations, requiring the use of juveniles that are even smaller. This restriction becomes a challenge when considering that optimizing microsatellites (SSRs) frequently requires multiple DNA samples, which is limited by the amount that can be extracted from each sample. Although the DNA quality of our samples exceeded expectations, we did get a lower number of reads from Novogene than originally expected. This should be considered by future researchers when defining the minimum coverage of their lcWGS.

Currently, several pipelines are available for identifying SSR markers from NGS data, including MISA [27], GMATA [28], and Micro-Primers [24]. For example, Micro-Primers is designed to generate primers from multiplexed libraries that contain pooled individuals. The authors suggest that pooling samples in this way can significantly reduce the cost of developing microsatellites for non-model species. While we agree that this approach can be more cost-effective, we selected the MiMi pipeline because it processes each individual separately during SSR detection. This strategy allows researchers to identify loci that are not only polymorphic but also consistently present across multiple individuals, which increases the likelihood that the markers will amplify successfully in downstream applications. Additionally, programs such as MISA [27] and GMATA [28] are not optimized for handling paired-end NGS data, as previously discussed by Alves et al. [24]. These tools require conversion of reads into FASTA format, which results in the loss of important pairing information critical for accurate SSR detection. Given these limitations, we chose the MiMi pipeline, and our experimental testing on field-collected individuals confirms that this method produces SSR markers that are reliable and usable—unlike other pipelines which did not test their developed markers on field-collected samples. However, our experimental test showed that some markers had less than three alleles and some markers failed to amplify in our samples. These factors should be considered when using this pipeline.

Our experimental test of the primers on two natural populations of *T. evexum* showed that most loci had low genetic diversity (average Ho = 0.478) and a deficit of heterozygotes (F = 0.226). Few studies have evaluated genetic diversity in tropical spiders, and most available research is limited to marker development with basic diversity estimates. Studies on *Paratrechalea galianoae* and *P. ornata* (Araneae: Trechaleidae) from South American populations reported higher levels of genetic diversity than those we found in *T. evexum* [15–17]. The only comparable estimate was reported by Macrini et al. [15] for *Aglaoctenus lagotis* (Araneae: Lycosidae) in Brazil, but the authors attributed the reduced diversity to a high incidence of null alleles, while in our case only one locus showed signs of null alleles. These previous studies sampled spiders across broader spatial ranges and likely from larger populations. *T. evexum* has small populations that are restricted to mature and old secondary forests nearby water sources such as streams and permanent small ponds within a narrow elevational range [29]. This species appears to have low juvenile dispersal, as juvenile abundance is higher in areas where females from the previous generation reproduced. Their populations have further been reduced and their isolation increased by urbanization and habitat loss. Limited gene flow and increased inbreeding in spiders appear to be widespread consequences of both natural and anthropogenic habitat fragmentation. For instance, the wolf spider *Rabidosa rabida* showed a significant increase in population homozygosity as populations became isolated due to land-use changes [36]. Additionally, studies of spiders inhabiting more natural environments revealed that population divergence, based on the mitochondrial cytochrome oxidase c subunit I, varied greatly among species [37]. The synergetic effect of these factors may explain the lower levels of genetic diversity and high levels of inbreeding we detected in *T. evexum* natural populations, possibly because of increasing consanguineous matings and genetic drift. However, given that we analyzed only 15 individuals across two populations, limited sample sizes may also influence our results. We are currently conducting an additional study with larger sample sizes to better understand how fragmentation and expansion of the urbanscape affect genetic diversity and gene flow patterns in this species.

We consider that MiMi script is a good alternative to develop primers for SSR markers for species without any previous information. The script is user-friendly and processes information from multiple individuals simultaneously, which allows for the identification of polymorphic markers. Using this protocol, we were able to recover 32 putative loci, with an approximate cost of US$2000 (laboratory costs only). Compared to previous methods [38], where costs exceeded US$10000. There are yet a few aspects that can be improved to make the MiMi script more user-friendly, which have been previously recognized by other authors [24]. The initial *pal_finder* steps that are performed on the free Galaxy server require a long time to run and are limited by hard disk space (50 GB). For our samples, we had to create three different accounts to be able to process the sequences from eight individuals. The MiMi script was run on an HPC computer; therefore, including the *pal_finder* analysis as part of the MiMi script would be more efficient, as it would not be constrained by the restrictions of the Galaxy platform. Finally, the MiMi manual should provide an estimate of the disk space required to complete the analysis. In our case, MiMi required more than 100 GB of space, which may be more than what HPC users typically get allotted. Knowing how much hard drive space is needed for the analysis avoids potential delays due to storage shortages. Our experimental test of the 13 most polymorphic loci on sampled individuals revealed that about 10% of the primers did not amplify, so researchers should anticipate that a small percentage (approx. 10%) of the loci identified by MiMi may fail in experimental tests. Nonetheless, this remains a cost-effective method for developing SSR markers on understudied tropical species.

## Supporting information

S1 File DNA extraction in *Theridion evexum.* (PDF)

S2 File Pal_finder protocol in Galaxy and MiMi protocol. (PDF)

S1 Table Number of microsatellites identified for *Theridion evexum* at each filtering stage, categorized by repeat motif. (PDF)

S2 Table Primer sequences (forward and reverse), *in silico* number of alleles, and allele repeat motif for 34 microsatellite loci isolated from *T. evexum* (Araneae: Theridiidae), using lcWGS and the MiMi bioinformatics pipeline. Primers in bold font were tested experimentally.
(PDF)

## References

[1] Biamonte E, Sandoval L, Chacón E, Barrantes G. Effect of urbanization on the avifauna in a tropical metropolitan area. Landsc Ecol. 2010;26(2):183–94. doi: 10.1007/s10980-010-9564-0

[2] Fuchs EJ, Hamrick JL. Spatial genetic structure within size classes of the endangered tropical tree Guaiacum sanctum (Zygophyllaceae). Am J Bot. 2010;97(7):1200–7. doi: 10.3732/ajb.0900377 21616871

[3] Rodríguez-Bardía M, Fuchs EJ, Barrantes G, Madrigal-Brenes R, Sandoval L. Genetic structure in neotropical birds with different tolerance to urbanization. Sci Rep. 2022;12(1):6054. doi: 10.1038/s41598-022-09961-9 35411055 PMC9001702

[4] Burkman CE, Gardiner MM. Urban greenspace composition and landscape context influence natural enemy community composition and function. Biological Control. 2014;75:58–67. doi: 10.1016/j.biocontrol.2014.02.015

[5] Corcos D, Cerretti P, Caruso V, Mei M, Falco M, Marini L. Impact of urbanization on predator and parasitoid insects at multiple spatial scales. PLoS One. 2019;14(4):e0214068. doi: 10.1371/journal.pone.0214068 30943220 PMC6447152

[6] Bonte D, Vandenbroecke N, Lens L, Maelfait J-P. Low propensity for aerial dispersal in specialist spiders from fragmented landscapes. Proc Biol Sci. 2003;270(1524):1601–7. doi: 10.1098/rspb.2003.2432 12908981 PMC1691421

[7] Fenoglio MS, Rossetti MR, Videla M. Negative effects of urbanization on terrestrial arthropod communities: A meta‐analysis. Global Ecol Biogeogr. 2020;29(8):1412–29. doi: 10.1111/geb.13107

[8] Piano E, Giuliano D, Isaia M. Islands in cities: Urbanization and fragmentation drive taxonomic and functional variation in ground arthropods. Basic and Applied Ecology. 2020;43:86–98. doi: 10.1016/j.baae.2020.02.001

[9] Delaney KS, Riley SPD, Fisher RN. A rapid, strong, and convergent genetic response to urban habitat fragmentation in four divergent and widespread vertebrates. PLoS One. 2010;5(9):e12767. doi: 10.1371/journal.pone.0012767 20862274 PMC2940822

[10] Lapinski W, Tschapka M. Desiccation resistance reflects patterns of microhabitat choice in a Central American assemblage of wandering spiders. J Exp Biol. 2014;217(Pt 15):2789–95. doi: 10.1242/jeb.102533 24855682

[11] Concepción ED, Moretti M, Altermatt F, Nobis MP, Obrist MK. Impacts of urbanisation on biodiversity: the role of species mobility, degree of specialisation and spatial scale. Oikos. 2015;124(12):1571–82. doi: 10.1111/oik.02166

[12] Hamilton CA, Lemmon AR, Lemmon EM, Bond JE. Expanding anchored hybrid enrichment to resolve both deep and shallow relationships within the spider tree of life. BMC Evol Biol. 2016;16(1):212. doi: 10.1186/s12862-016-0769-y 27733110 PMC5062932

[13] Liu J, May-Collado LJ, Pekár S, Agnarsson I. A revised and dated phylogeny of cobweb spiders (Araneae, Araneoidea, Theridiidae): A predatory Cretaceous lineage diversifying in the era of the ants (Hymenoptera, Formicidae). Mol Phylogenet Evol. 2016;94(Pt B):658–75. doi: 10.1016/j.ympev.2015.09.023 26454029

[14] Moyle RG, Taylor SS, Oliveros CH, Lim HC, Haines CL, Rahman MA, et al. Diversification of an endemic Southeast Asian genus: Phylogenetic relationships of the spiderhunters (Nectariniidae:Arachnothera). The Auk. 2011;128(4):777–88. doi: 10.1525/auk.2011.11019

[15] Cryptic diversity of Aglaoctenus lagotis (Araneae, Lycosidae) in the Brazilian Atlantic Rainforest: evidence from microsatellite and mitochondrial DNA sequence data. J App Biol Biotech. 2015. doi: 10.7324/jabb.2015.3602

[16] Pandulli-Alonso I, Germil M, Albo MJ, Tomasco IH. Characterization of four hypervariable microsatellite loci in a nuptial gift-giving spider and its prospect for paternity analyses. Arachnology. 2020;18(5):477. doi: 10.13156/arac.2020.18.5.477

[17] DA Silveira LCT, Bonatto SL. Isolation and characterization of 12 dinucletiotide microsatellite loci in Paratrechalea galianoae (Araneae, Trechaleidae), a nuptial gift-spider. Mol Ecol Resour. 2009;9(2):539–41. doi: 10.1111/j.1755-0998.2008.02338.x 21564686

[18] Bhargava A, Fuentes FF. Mutational dynamics of microsatellites. Mol Biotechnol. 2010;44(3):250–66. doi: 10.1007/s12033-009-9230-4 20012711

[19] Rose O, Falush D. A threshold size for microsatellite expansion. Mol Biol Evol. 1998;15(5):613–5. doi: 10.1093/oxfordjournals.molbev.a025964 9580993

[20] Watts AG, Schlichting PE, Billerman SM, Jesmer BR, Micheletti S, Fortin M-J, et al. How spatio-temporal habitat connectivity affects amphibian genetic structure. Front Genet. 2015;6:275. doi: 10.3389/fgene.2015.00275 26442094 PMC4561841

[21] Yañez-Yazlle MF, Broglia VG, Caruso GB. Transferibilidad de marcadores SSR de tomate (Solanum lycopersicum) al chilto (Solanum betaceum) para la evaluación de la diversidad genética de dos poblaciones del Noroeste Argentino. Lhawet. 2022;8.

[22] Fuchs EJ, Cascante-Marín A, Madrigal-Brenes R, Quesada M. Genetic diversity and phylogeographic patterns of the dioecious palm Chamaedorea tepejilote (Arecaceae) in Costa Rica: the role of mountain ranges and possible refugia. AoB Plants. 2022;15(1):plac060. doi: 10.1093/aobpla/plac060 36654989 PMC9840212

[23] Abdelkrim J, Robertson B, Stanton J-A, Gemmell N. Fast, cost-effective development of species-specific microsatellite markers by genomic sequencing. Biotechniques. 2009;46(3):185–92. doi: 10.2144/000113084 19317661

[24] Alves F, Martins FMS, Areias M, Muñoz-Mérida A. Automating microsatellite screening and primer design from multi-individual libraries using Micro-Primers. Sci Rep. 2022;12(1):295. doi: 10.1038/s41598-021-04275-8 34997147 PMC8741888

[25] Fox G, Preziosi RF, Antwis RE, Benavides-Serrato M, Combe FJ, Harris WE, et al. Multi-individual microsatellite identification: A multiple genome approach to microsatellite design (MiMi). Mol Ecol Resour. 2019;19(6):1672–80. doi: 10.1111/1755-0998.13065 31339632 PMC6900094

[26] Griffiths SM, Fox G, Briggs PJ, Donaldson IJ, Hood S, Richardson P, et al. A Galaxy-based bioinformatics pipeline for optimised, streamlined microsatellite development from Illumina next-generation sequencing data. Conserv Genet Resour. 2016;8(4):481–6. doi: 10.1007/s12686-016-0570-7 32355508 PMC7175698

[27] Thiel T, Michalek W, Varshney RK, Graner A. Exploiting EST databases for the development and characterization of gene-derived SSR-markers in barley (Hordeum vulgare L.). Theor Appl Genet. 2003;106(3):411–22. doi: 10.1007/s00122-002-1031-0 12589540

[28] Wang X, Wang L. GMATA: An Integrated Software Package for Genome-Scale SSR Mining, Marker Development and Viewing. Front Plant Sci. 2016;7:1350. doi: 10.3389/fpls.2016.01350 27679641 PMC5020087

[29] Barrantes G, Weng JL. Natural History, Courtship, Feeding Behaviour and Parasites ofTheridion evexum(Araneae: Theridiidae). Arachnology. 2007;14(2):61–5. doi: 10.13156/arac.2007.14.2.61

[30] Gregory TR, Shorthouse DP. Genome sizes of spiders. J Hered. 2003;94(4):285–90. doi: 10.1093/jhered/esg070 12920099

[31] Bolger AM, Lohse M, Usadel B. Trimmomatic: a flexible trimmer for Illumina sequence data. Bioinformatics. 2014;30(15):2114–20. doi: 10.1093/bioinformatics/btu170 24695404 PMC4103590

[32] Castoe TA, Poole AW, de Koning APJ, Jones KL, Tomback DF, Oyler-McCance SJ, et al. Rapid microsatellite identification from Illumina paired-end genomic sequencing in two birds and a snake. PLoS One. 2012;7(2):e30953. doi: 10.1371/journal.pone.0030953 22348032 PMC3279355

[33] Liao P, Satten GA, Hu Y-J. PhredEM: a phred-score-informed genotype-calling approach for next-generation sequencing studies. Genet Epidemiol. 2017;41(5):375–87. doi: 10.1002/gepi.22048 28560825 PMC5564424

[34] Van Oosterhout C, Hutchinson WF, Wills DPM, Shipley P. micro‐checker: software for identifying and correcting genotyping errors in microsatellite data. Molecular Ecology Notes. 2004;4(3):535–8. doi: 10.1111/j.1471-8286.2004.00684.x

[35] R Core Team. R: A language and environment for statistical computing. Vienna, Austria: R Foundation for Statistical Computing; 2024. Available: http://www.R-project.org

[36] Reed DH, Teoh V-H, Stratton GE, Hataway RA. Levels of gene flow among populations of a wolf spider in a recently fragmented habitat: current versus historical rates. Conserv Genet. 2009;12(1):331–5. doi: 10.1007/s10592-009-9995-9

[37] Botham JL, Haddad CR, Gryzenhout M, Swart VR, Bredenhand E. High genetic diversity of spider species in a mosaic montane grassland landscape. PLoS ONE. 2020;15(6):e0234437. doi: 10.1371/journal.pone.0234437 PMC7279597 32511281

[38] Fuchs EJ, Cascante-Marin A, Madrigal-Brenes R, Harvey N, Quesada M. Isolation and characterization of microsatellites for the neotropical dioecious palm Chamaedorea tepejilote (Arecaceae) and cross-amplification in other Chamaedorea species. Mol Biol Rep. 2020;47(8):6385–91. doi: 10.1007/s11033-020-05580-7 32557191

