# Supplementary material for: Microsatellite development for Theridion evexum (Araneae: Theridiidae) using low-coverage genome sequencing and the MiMi script
Source: PLoS One. 2025 Sep 15;20(9):e0331200. doi: 10.1371/journal.pone.0331200 (PMC12435640; doi:10.1371/journal.pone.0331200)
Supplement: S1 File — (PDF) [file pone.0331200.s001.pdf]

protocols.io

Jan 03, 2025

DNA extraction in *Theridion evexum*

DOI
dx.doi.org/10.17504/protocols.io.kxygxwp2kv8j/v1

Ruth Madrigal-Brenes[1], Gilbert Barrantes[1], Luis Sandoval[1], Eric J. Fuchs[1]
[1]Universidad de Costa Rica

Eric J. Fuchs
Universidad de Costa Rica

OPEN ACCESS

DOI: dx.doi.org/10.17504/protocols.io.kxygxwp2kv8j/v1

Protocol Citation: Ruth Madrigal-Brenes, Gilbert Barrantes, Luis Sandoval, Eric J. Fuchs 2025. DNA extraction in Theridion evexum. **protocols.io** https://dx.doi.org/10.17504/protocols.io.kxygxwp2kv8j/v1

License: This is an open access protocol distributed under the terms of the **Creative Commons Attribution License**, which permits unrestricted use, distribution, and reproduction in any medium, provided the original author and source are credited

Protocol status: Working
We use this protocol and it's working

Created: November 22, 2024

Last Modified: January 03, 2025

Protocol Integer ID: 112656

Keywords: microsatellites, tropical spiders, CTAB, Costa Rica

Funders Acknowledgements:
Vicerrectoría de Investigación
Grant ID: C3-116

Abstract

The number of species threatened by fragmentation and loss of natural habitats due to changes in land use and unplanned urban expansion are rapidly increasing. Despite the seriousness of the situation, information on the impact of isolation caused by fragmentation and urbanization on spider genetic diversity is scarce, owing mostly to a lack of appropriate molecular markers. The main objective of this study was to develop microsatellite (SSR) primers for the spider Theridion evexum using low-coverage next-generation sequencing and bioinformatic tools. To increase the yield of DNA extracted from small spiders like T. evexum, we also optimized a CTAB DNA extraction protocol. We sequenced eight individuals at 4X using paired-end sequencing on an Illumina Novaseq 6000. Reads were cleaned and processed using the MiMi python pipeline. MiMi produced a total of 3999 putative microsatellite primers. After filtering for polymorphic loci with an allelic richness greater than three and primers that were present in at least 5 of the 8 sequenced individuals, 34 final markers were identified. An in vivo test of 13 of these 34 markers showed that 10 loci were polymorphic with at least three detectable alleles, one locus was monomorphic, and two loci did not produce PCR products. These markers will allow a better assessment of the effects of fragmentation and isolation across populations of this spider species. Furthermore, developing markers using low-coverage NGS (next-generation sequencing) and bioinformatic methods provide a valuable approach for uncovering SSR markers at a reduced cost for other tropical species, thereby broadening the scope of molecular ecology research in the tropics.

Protocol materials

- Liquid nitrogen
- Polyvinylpyrrolidone (PVP-40) **Bio Basic Inc. Catalog #**PB0436.SIZE.250g
- EDTA disodium dihydrate **Abblis Catalog #**AB1011793
- CTAB
- Sodium Chloride **Catalog #**PubChem CID: 5234
- Proteinase K (20 mg/ml)
- Chlorofom-octanol (24:1)
- Isopropanol
- TE Buffer
- 70% Ethanol

Sample preparation

1 Remove the sample from the alcohol in which it was preserved and place it in a Petri
dish under the stereoscope.

2 Remove the sample's abdomen and discard it, leaving only the cephalothorax and legs.

3 Place the cephalothorax and legs (called from now on "the sample") on a piece of
paper towel to remove as much alcohol as possible from the sample.

4 Leave the sample at room temperature for 5 minutes to allow any remaining alcohol to
evaporate.

DNA extraction

3h 24m 5s

5 Transfer the sample to a pre-chilled (with liquid nitrogen for 15 seconds) 1.5 mL microcentrifuge tube.

6 Add approximately 1 mL Liquid nitrogen **Contributed by users** to freeze the sample completely.

7 Ground the sample into small pieces (< 1 mm) or a fine powder using a plastic pestle.

8 Add 500 µL CTAB buffer ([M] 2 % volume CTAB: [M] 100 millimolar (mM) [M] 1.4 Molarity (M) Sodium Chloride **Contributed by users Catalog** #PubChem CID: 5234 , [M] 20 millimolar (mM) EDTA disodium dihydrate **Abblis Catalog** #AB1011793 , [M] 2 Mass / % volume CTAB **Contributed by users** , [M] 2 % (v/v) Polyvinylpyrrolidone (PVP-40) **Bio Basic Inc. Catalog** #PB0436.SIZE.250g
)

9 Add 20 µL of Proteinase K (20 mg/ml) **Contributed by users**

10 Incubate Overnight at 55 °C with continuous shaking at 800 rpm.

11 1. Centrifuge samples 14000 rpm, Room temperature, 00:05:00 5m

12 Transfer 400 µL of the supernatant to a 2 mL tube.

13 Add 400 µL of Chlorofom-octanol (24:1)

14 Mix by inverting the tubes for 00:02:00 2m

15 Centrifuge samples 14000 rpm, Room temperature, 00:05:00 5m

16 Transfer 300 µL of the supernatant (without disturbing the organic phase) to a 1.5 mL tube.

17 Add 300 µL of Isopropanol **Contributed by users** at -20 °C .

18 Mix by inverting the tubes for 00:02:00 . 2m

19 Place the samples on ice and refrigerate at -20 °C for 01:00:00 (or overnight). 1h

20 Centrifuge samples 14000 rpm, Room temperature, 00:05:00 5m

21 Discard all the supernatant ensuring not to disturb the pellet (important: remove supernatant as much as you can).

22 Add 500 µL 70% Ethanol **Contributed by users** .

23 Mix by vortex for 00:00:05 . 5s

24 Centrifuge samples 14000 rpm, Room temperature, 00:05:00 — 5m

25 Discard all the ethanol by decantation ensuring not to disturb the pellet (important: remove alcohol as much as you can).

26 Repeat steps 22-25 at least once again.

27 Leave the tubes upside down on a napkin to remove excess ethanol for 02:00:00 — 2h

28 Once the pellet was completely dry, it was eluted in 50 µL of TE Buffer **Contributed by users** .

protocols.io
