# Supplementary material for: Microsatellite development for Theridion evexum (Araneae: Theridiidae) using low-coverage genome sequencing and the MiMi script
Source: PLoS One. 2025 Sep 15;20(9):e0331200. doi: 10.1371/journal.pone.0331200 (PMC12435640; doi:10.1371/journal.pone.0331200)
Supplement: S2 File — (PDF) [file pone.0331200.s002.pdf]

protocols.io

Jan 03, 2025

# Pal_finder protocol in Galaxy and MiMi protocol

DOI
**dx.doi.org/10.17504/protocols.io.dm6gp9bo8vzp/v1**

Ruth Madrigal-Brenes[1], Gilbert Barrantes[1], Luis Sandoval[1], Eric J. Fuchs[1]
[1]Universidad de Costa Rica

Ruth Madrigal-Brenes
Universidad de Costa Rica

OPEN ACCESS

**DOI:** **dx.doi.org/10.17504/protocols.io.dm6gp9bo8vzp/v1**

**Protocol Citation:** Ruth Madrigal-Brenes, Gilbert Barrantes, Luis Sandoval, Eric J. Fuchs 2025. Pal_finder protocol in Galaxy and MiMi protocol. **protocols.io** **https://dx.doi.org/10.17504/protocols.io.dm6gp9bo8vzp/v1**

**License:** This is an open access protocol distributed under the terms of the **Creative Commons Attribution License**, which permits unrestricted use, distribution, and reproduction in any medium, provided the original author and source are credited

**Protocol status:** Working
**We use this protocol and it's working**

**Created:** November 29, 2024

**Last Modified:** January 03, 2025

**Protocol Integer ID:** 113161

**Keywords:** microsatellites, bioinformatics, tropical spider, Costa Rica, MiMi

**Funders Acknowledgements:**
**Vicerrectoría de Investigación**
Grant ID: C3-116

## Abstract

The number of species threatened by fragmentation and loss of natural habitats due to changes in land use and unplanned urban expansion are rapidly increasing. Despite the seriousness of the situation, information on the impact of isolation caused by fragmentation and urbanization on spider genetic diversity is scarce, owing mostly to a lack of appropriate molecular markers. The main objective of this study was to develop microsatellite (SSR) primers for the spider Theridion evexum using low-coverage next-generation sequencing and bioinformatic tools. To increase the yield of DNA extracted from small spiders like T. evexum, we also optimized a CTAB DNA extraction protocol. We sequenced eight individuals at 4X using paired-end sequencing on an Illumina Novaseq 6000. Reads were cleaned and processed using the MiMi python pipeline. MiMi produced a total of 3999 putative microsatellite primers. After filtering for polymorphic loci with an allelic richness greater than three and primers that were present in at least 5 of the 8 sequenced individuals, 34 final markers were identified. An in vivo test of 13 of these 34 markers showed that 10 loci were polymorphic with at least three detectable alleles, one locus was monomorphic, and two loci did not produce PCR products. These markers will allow a better assessment of the effects of fragmentation and isolation across populations of this spider species. Furthermore, developing markers using low-coverage NGS (next-generation sequencing) and bioinformatic methods provide a valuable approach for uncovering SSR markers at a reduced cost for other tropical species, thereby broadening the scope of molecular ecology research in the tropics.

## Set up

1 Create a user on **https://palfinder.ls.manchester.ac.uk/**.

2 Upload the raw sequences of each individual.

## Sequence Quality Verification

3 Check sequences using FastQC with the following configuration:

3.1 **Short read data**: Select raw sequences, both Forward" (R1) and Reverse (R2), separately for each individual.

3.2 **Contaminant list**: None.

3.3 **Adapter list**: Upload a text file (.txt) containing adapter names and sequences.

3.4 **Submodule and limit specifying file**: None.

3.5 **Disable grouping of bases for reads >50 bp**: None.

3.6 **Lower limit on sequence length**: Default setting (blank).

3.7 **Length of Kmer**: Default setting (7).

## Sequence Cleaning

4 Clean sequences using Trimmomatic-Galaxy Version 0.38.1 with the following configuration:

4.1 **Single-end or paired-end reads?** Select paired-end (two separate input files).

4.2 **Perform initial ILLUMINACLIP step?** Yes.

4.3 **Select standard adapter sequences or provide custom?** Select Custom.

4.4 **Paste Customized adapter sequences in FASTA format.**

4.5 **Maximum mismatch count which will still allow a full match to be performed:** 2

4.6 **How accurate the match between the two 'adapter ligated' reads must be for PE palindrome read alignment:** 30.

4.7 **How accurate the match between any adapter etc. sequence must be against a read:** 10

4.8 **Minimum length of adapter that needs to be detected (PE specific/palindrome mode):** 8.

4.9 **Always keep both reads (PE specific/palindrome mode)?** Yes

4.10 **SLIDINGWINDOW:** 4, 30

4.11 **LEADING:** 3

4.12 **TRAILING:** 3

4.13 **MINLEN**: 50

4.14 **Output trimlog file?** Yes.

4.15 **Output trimmomatic log messages?** Yes.

4.16 Save the outputs

## Individual Microsatellite Identification

5 Run pal_finder in the Galaxy server with the following configuration:

5.1 **Primer prefix**: Enter a text prefix that will identify each individual.

5.2 **Sequencing platform used to generate data**: Select the Illumina option.

5.3 **Input Type**: Chose the "Pair of datasets" option. Select the paired sequences obtained with Trimmomatic (outputs of step 4.16).

5.4 **Use all reads for microsatellite detection?** Yes, as all sequences had been filtered using Trimmomatic.

5.5 **Filters to apply to the pal_finder results**: Selected all the default options.

5.6 **Use PANDAseq to assemble paired-end reads and confirm primer sequences are present in high-quality assembly**: Yes.

5.7 **Minimum number of 2-mer repeat units to detect**: 6

5.8 **Minimum number of 3-mer repeat units**: 4

5.9 **Minimum number of 4-mer, 5-mer, and 6-mer repeat units**: 3

5.10 **Mispriming library to use**: Default from pal_finder

5.11 **Primer settings to use**: Default from pal_finder (except for the minimum and maximum GC percentage. Set it within a range of 40–60%).

## SSR´s development with MiMi

6 Create a user account on an HPC cluster and ensure sufficient storage space (approximately 100 GB).

7 Download the MiMi program (https://github.com/graemefox/mimi).

8 Load the necessary modules for the program's functionality (Biopython, PANDAseq, MUSCLE, MiMi).

9 Upload the cleaned sequences for each individual and decompress them on the HPC cluster.

10 Edit the MiMi configuration file to adjust paths to the MiMi program location and the files uploaded in step 9.

11 Run MiMi. with the follosing command:

```
python MiMi_v0.1.2.py -c 'path to configuration file'
```

12 Download the output file named: MiMi_output_all_loci

## SSR´s filtration

13 Open the file **MiMi_output_all_loci o**n an any spreed sheet software.

14 Filter monomorphic microsatellites by filtering the column “Size_Range” to loci that have a value greater than 0.

15 From the potential polymorphic microsatellites, filter those present in at least 6 of the 8 analyzed individuals (75% of the samples) by selecting the column named “Samples_containing_raw_reads_(respectively)” with 6 or more samples. And allergic richness of Ar = 3 or higher, by filtering the column “Alleles_present‘ with three or more alleles.
