# Supplementary material for: Microsatellite development for Theridion evexum (Araneae: Theridiidae) using low-coverage genome sequencing and the MiMi script
Source: PLoS One. 2025 Sep 15;20(9):e0331200. doi: 10.1371/journal.pone.0331200 (PMC12435640; doi:10.1371/journal.pone.0331200)
Supplement: S1 Table — (PDF) [file pone.0331200.s003.pdf]

Supplemental Table 1. Number of microsatellites identified for *Theridion evexum* at each filtering stage, categorized by repeat motif.

**Step**	**Dinucleotide**	**Trinucleotide**	**Tetranucleotide**	**Pentanucleotide**	**Hexanucleotide**	**Total**
SSRs identified by MiMi	1135	891	1658	294	21	3999
After filtering for polymorphism (more than 2 alleles per marker)	253	192	97	12	1	555
Markers present on 5 or more individuals with more than 3 alleles	25	5	4	0	0	34
Markers present on 6 or more individuals with more than 3 alleles	10	3	0	0	0	13
