# Supplementary material for: Microsatellite development for Theridion evexum (Araneae: Theridiidae) using low-coverage genome sequencing and the MiMi script
Source: PLoS One. 2025 Sep 15;20(9):e0331200. doi: 10.1371/journal.pone.0331200 (PMC12435640; doi:10.1371/journal.pone.0331200)
Supplement: S2 Table — Primers in bold font were tested experimentally. (PDF) [file pone.0331200.s004.pdf]

Supplemental Table 2. Primer sequences (forward and reverse), *in silico* number of alleles, and allele repeat motif for 34 microsatellite loci isolated from *T. evexum* (Araneae: Theridiidae), using lcWGS and the MiMi bioinformatics pipeline. Primers in bold font were tested experimentally.

Primer	Forward Sequence (5'- 3')	Reverse Sequence (5'- 3')	No. Alleles *in silico*	Repeat motif
**Thev01**	TGTTAATGCTGAAAGACGCC	TTGCTGAAGGGTATCTTAACGC	3	AT
**Thev02**	ACATTTCAGTGCTTCCAGTAACC	AGCAAATGCTGTGTTCTGCC	3	ATT
**Thev03**	TGCACCAATAAAAGGGGTCC	TCAGGATCAGACCATTTCAGG	3	TC
**Thev04**	TTTCCGTATTCAACTACTTAGCCG	CGGAATTTGTTTTTTTTGGTTCGG	3	AGT
**Thev05**	CTTGACCGTATTGCGCGCAGC	GTCTTGTGTGCATTGCATTCCC	3	ATT
**Thev06**	TTGCGACCCTTGTAAAGACC	TATGTAGGATGTTCCCTTTTGC	3	AC
**Thev07**	TCTGAGTTTCTCAAATCAACCCC	CCAGGGGGCTCATATCTTATTATTCTTGC	3	AC
**Thev08**	CGAGATGTTTAGCTCCTTCTTCTGC	GAATATCGTTTTTTCTCCGCCGCCC	3	AT
**Thev09**	TGCAGTTGCTACGCTACGCTACAGG	GAGTTATTTTCGTGTGGAAGCCG	4	TC
**Thev10**	CCCTTAGGCCAACTAACCTCG	TGCATTGGAGAAAACTTTCGG	3	TC
**Thev11**	CTTATGCAATGGGAAAGGGC	ATTTATTCCCGGTTCCTCCC	3	TC
**Thev12**	CTCGTGCAACGAAAATGAGG	GGTTACGATCCAACCCTGC	3	AT
**Thev13**	TTAAGTTTTTGGAGAACGGGGGGC	TGCATTAGGACCGGCATAGC	4	AT
Thev14	TATCGTGCAATGTTGTGGGC	GCAATTCGAAAGAAGAGCGG	3	TC
Thev15	TTACTGAAACATGGGGGGCAACC	CATTGAAAGTCTGGTTTTTCTGATCG	3	AT
Thev16	TTCACACACTGGGAGTGTTGGG	AAATTGGTTGCCCTTATCGG	3	TC
Thev17	GAGCAGAAGTGGAAGGATGG	CCATAACTTAGAATATATCGACCGC	3	ATT
Thev18	CCCCACACATTCAATCCTGGC	CATTGGAATAAATCCATTCAGGAGC	3	TC
Thev19	TTTGGGGCAAACATAAGTGCCG	CAATGTGTTACTGCAGATAATGGC	3	TC
Thev20	CCAAAGTTACATGCAGCAGCCATAGG	ATCGCGTCCAACCTCTCTGACC	3	TC
Thev21	TTGGATAGAGCTGACTCATTTCCG	GGCATAACAAACATCAGAGAGGC	3	TC
Thev22	GCTTGCACTTTTGGTTTTTTTTGC	ACTACAAGCATACATTACTCTCTGCTCC	3	TC
Thev23	GGGTTCAGTCAGTGACAAATATATACG	CACAAAATGCATACCATCAGTTCC	3	AT

Thev24	CACTCATGTTTAATGAATCCCCCC	GCATTAACTGCATGCATATGGATGG	3	AC
Thev25	TCCGTTGTTGTTCTTACTACCACAACC	TACCCAATTTTCGGGCTAGG	3	AT
Thev26	TTGTTACGTCTTGACATTACCGC	TACCAAAGACTCGCGAATGG	3	AAAT
Thev27	AAGGGAAAGTTGGGAGTGAGC	GTCGTTATTAGGGCATCGGC	3	AAAT
Thev28	GAAAGCTGTTCATGGTGTTTGTTTCG	TGCTTGTCTCTTAGCAAATGCG	3	AAAT
Thev29	CTGTTGCAAGTGCCATCG	TGAAGTCAGAGAAACGACCTGG	3	TC
Thev30	CCTCAAACATTTCTCAAAGACTGC	GATTAATATATCCAATCCTTTCCGAGG	4	TC
Thev31	TCTACTCTCTATCGCCGTGCTCC	TGGCAGAAATAAGTTCCGTGG	4	AC
Thev32	TGCCTTTTCGATAATCTGTGTGC	CGAAGCTAGAATTCTCTCATTACAGCC	4	ATT
Thev33	GTTAACACTAAATTCTCTTGCCGC	TGTCGATGGCTAACTGCG	5	AT
Thev34	CTCACAAAAGCTGTATCAAAAGCC	CAAGTAGTAAAACTATATCCTCCTACGGC	5	AAAG
